# A Quantitative Approach to the Prioritization of Zoonotic Diseases in North America: A Health Professionals’ Perspective

**DOI:** 10.1371/journal.pone.0072172

**Published:** 2013-08-21

**Authors:** Victoria Ng, Jan M. Sargeant

**Affiliations:** Centre for Public Health and Zoonoses, Department of Population Medicine, Ontario Veterinary College, University of Guelph, Guelph, Canada; Université Catholique de Louvain, Belgium

## Abstract

**Background:**

Currently, zoonoses account for 58% to 61% of all communicable diseases causing illness in humans globally and up to 75% of emerging human pathogens. Although the impact of zoonoses on animal health and public health in North America is significant, there has been no published research involving health professionals on the prioritization of zoonoses in this region.

**Methodology/Principal Findings:**

We used conjoint analysis (CA), a well-established quantitative method in market research, to identify the relative importance of 21 key characteristics of zoonotic diseases for their prioritization in Canada and the US. Relative importance weights from the CA were used to develop a point-scoring system to derive a recommended list of zoonoses for prioritization in Canada and the US. Study participants with a background in epidemiology, public health, medical sciences, veterinary sciences and infectious disease research were recruited to complete the online survey (707 from Canada and 764 from the US). Hierarchical Bayes models were fitted to the survey data to derive CA-weighted scores for disease criteria. Scores were applied to 62 zoonotic diseases to rank diseases in order of priority.

**Conclusions/Significance:**

We present the first zoonoses prioritization exercise involving health professionals in North America. Our previous study indicated individuals with no prior knowledge in infectious diseases were capable of producing meaningful results with acceptable model fits (79.4%). This study suggests health professionals with some knowledge in infectious diseases were capable of producing meaningful results with better-fitted models than the general public (83.7% and 84.2%). Despite more similarities in demographics and model fit between the combined public and combined professional groups, there was more uniformity across priority lists between the Canadian public and Canadian professionals and between the US public and US professionals. Our study suggests that CA can be used as a potential tool for the prioritization of zoonoses.

## Introduction

Zoonotic diseases are diseases that are naturally transmitted between vertebrate animals and humans [1], [2]. Currently, it is estimated that zoonoses account for 58% to 61% of all communicable diseases causing illness in humans globally [3], [4] and up to 75% of emerging human pathogens [3]–[5]. Although zoonotic outbreaks are a significant burden of disease in North America, for example, outbreaks of West Nile virus, SARS, H1N1 influenza and Lyme disease in the past decade [6]–[9], there are limited resources available for their control and prevention making it necessary to prioritize diseases in order to allocate resources to those with the greatest impact. While there is consensus on the need to prioritize zoonoses, there are numerous challenges to the process. These include, the difficulty in comparing the overall public health impact of zoonoses when they vary greatly in incidence, clinical manifestations, control measures, transmission potential and socio-economic impact in humans and animals [10], [11]; the multiple stakeholders involved each with their own prioritization objectives and beliefs [12]; and the lack of agreement in prioritization methodologies [13]–[15]. These challenges limit the ability to establish a universally accepted priority list for zoonoses. Nonetheless, a number of studies have attempted to prioritize communicable diseases [13], [16]–[21] and more recently, zoonotic diseases [22]–[26], though the studies on zoonotic diseases have all been conducted in Europe.

Progress has been made towards the use of quantitative approaches to address the complexity of disease prioritization and to overcome constraints in traditional methods [13], [14], [21], [24], [25]. Although methodological approaches differ, prioritization typically follows a series of steps: (1) selecting a group of diseases/pathogens to prioritize; (2) identifying a list of measurable criteria to assess diseases/pathogens; (3) defining a range of levels for each criterion; (4) determining the relative importance for each level by assigning a weight or score; (5) assigning weights and/or scores by matching the level of each disease criterion to the select group of diseases/pathogens; (6) aggregating weights/scores to produce an overall score for each disease/pathogen; and (7) ranking diseases/pathogens by their overall score to derive a priority list. Current prioritization methods, including recent quantitative approaches, are limited by the requirement to produce arbitrary scores and subjective weights for disease criteria and their levels (step 4), these are typically derived from a simplified linear point-scoring system without weights [16]–[20] or by an expert Delphi panel who assign weights that are applied to a linear point-scoring system [13], [14], [21], [24], [26]. This self-explicated approach to deriving scores and weights can introduce subjective bias into the prioritization exercise. Further, the matrix approach to assigning scores and weights to each disease criterion separately makes the assumption that disease criteria are independent.

A novel quantitative approach to overcoming these specific limitations is Conjoint Analysis (CA). CA is a market research technique used in exploring consumer preferences [27]. It is gaining recognition in the last decade for its use in eliciting preferences in the healthcare setting [28]–[31]. The principle behind CA is that a product (goods or service) can be described by a set of characteristics and the extent to which an individual values a product is determined by the level of each of those characteristics and the combination of those characteristics together [28], [29], [32]. A CA study presents individuals with competing products containing both desirable and undesirable characteristics and forces the individual to state a preference, usually as a choice between products. In doing so, individuals make a trade-off between the desirable and undesirable characteristics in the products through their choices revealing the true value of each characteristic relative to each other. This preference elicitation method overcomes the need to assign arbitrary scores and subjective weights as relative weighted scores for each characteristic and their levels are derived from the choice data. Additionally, this approach forces individuals to consider multiple characteristics together; thus, criteria are not assumed to be independent from each other.

In the context of zoonoses, a disease can be treated as a product described by a set of disease criteria (characteristics), and the value of the disease can be determined by the level of each criteria and the combination of those criteria. While similar methods to CA such as Maximum Difference Scaling (MaxDiff) are available [32], zoonotic diseases are often complex requiring an understanding of preferences under a range of multiple characteristics and levels, thus the use of CA, that allows for the exploration of inter-relationships across numerous characteristics and levels, was considered a more appropriate tool. An additional benefit in using CA in disease prioritization is that by presenting zoonoses as a set of disease characteristics without identifying diseases, individuals are forced to prioritize based on science, eliminating potential biases associated with disease names. These can include biases arising from prioritizing diseases on the basis of professional awareness and/or personal gain, or from the potential fear of a disease name compared to a lesser-known disease. Finally, by presenting respondents with all the information to prioritize diseases, CA allows for wide social participation; this can include experts who may not be familiar with the full range of diseases. CA is similar to the methodology used in a recent study on the prioritization of emerging zoonoses in The Netherlands [25], although a different mathematical approach was used.

Previously, we presented on the novel use of CA to develop a point scoring system for disease criteria considered important in determining priority amongst individuals from the general public [33]. The current study describes the results of individuals identified as ‘health professionals’ - individuals with a background in medical and veterinary sciences, public health, epidemiology and infectious diseases. This paper will also compare the results between the public groups and the professional groups. The primary objective of this study is to present on the methodological approach used to prioritize zoonoses in North America, a secondary objective is to define the most important zoonoses in North America as identified using this approach.

## Materials and Methods

### Study Participants

The Research Ethics Board at the University of Guelph approved all aspects of this study. The target study participants were: epidemiologists, public health practitioners and policymakers in the human and animal health disciplines at the local, provincial/state and national level, academic and practicing physicians and veterinarians, infectious disease researchers, human and animal health laboratory microbiologists, pathologists and technicians and registered nurses. Thus, the study group included individuals in relevant professional disciplines that would provide some prior knowledge of infectious diseases.

Participants from both Canada and the US were recruited through email invitation. Email searches were conducted for academic and practicing physicians and veterinarians and public health representatives and policymakers at the local, provincial/state and federal level. Direct email invitations were endorsed and sent by professional associations. Web advertisements were placed in relevant publications and some individuals were recruited in person at provincial and national conferences (see S1 for full listing). An additional 125 Canadian physicians were recruited online using a healthcare panel through Research Now™ [34]; these were groups of pre-screened Canadian physicians who had expressed a willingness to participate in online surveys. Reminder emails were sent a week after the initial email invitation and three weeks after the initial email invitation. Recruitment commenced in November 2010 and was completed in January 2012. Sample size calculations were made using Sawtooth Software SSI Web v7 [35]; a minimum of 500 professionals per country was needed for this study.

Surveys were collected online and anonymously. All participants acknowledged an informed consent assuring confidentiality and the option to withdraw from participation without penalty. Sawtooth Software SSI Web v7 [35] was used to screen participants through a series of demographic questions prior to survey commencement. Participants were disqualified if they did not reside in North America or were not employed in one of the following fields: epidemiology, public health, medical sciences, veterinary sciences, infectious disease research, laboratory technician or nursing.

### Survey Development

The methods for criteria identification, disease selection, literature review, defining levels for disease criteria, survey development and administration were described previously [33]. Briefly, six focus groups were conducted using the nominal group technique to identify the disease criteria in the study [12]. A total of 21 criteria were selected to inform the CA experimental design. Each of these selected criteria could be quantitatively measured with scientific data in the literature [33]. A total of 62 existing and emerging zoonotic and enteric diseases was selected for the study on the basis of being nationally or internationally notifiable or identified as a priority [33]. Diseases exhibiting multiple forms (for example, acute/chronic, latent/active) were divided into separate syndromes and approximate proportions were assigned to each syndrome as informed by the literature. There were 117 separate disease syndromes identified from the 62 diseases. A literature search for each criterion for each disease syndrome was conducted, searches included websites of reputable human and animal health organizations, reference textbooks and PubMed catalogued peer-reviewed publications [33]. Criterion levels were defined according to the range exhibited in the literature with three or four levels assigned to each criterion.

### Survey Instrument

**Figure 1:**
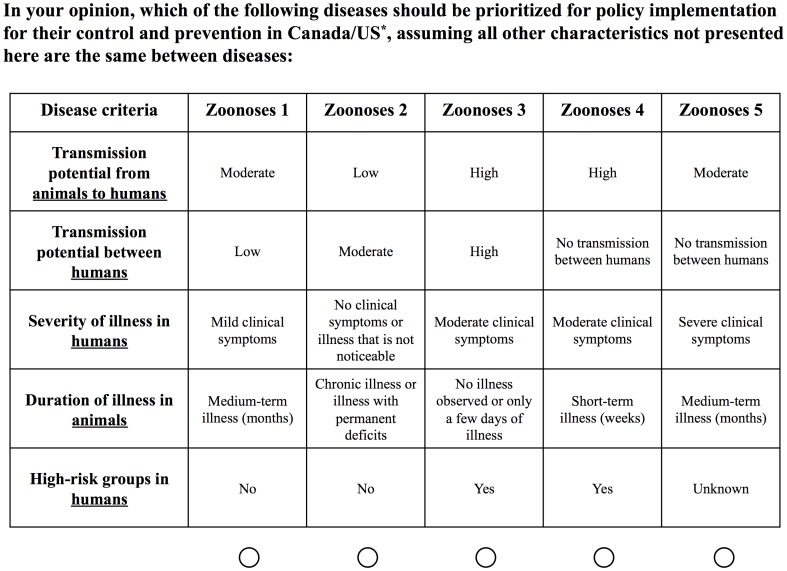
Example of one choice task set completed by each study participant. As multiple survey versions were administered randomly to each person, a different combination of disease criteria and levels was presented to study participants. The ordering of the presentation of disease criteria within each choice task was randomized to reduce ordering bias. (*Canadian participants were asked to prioritize for Canada while US participants were asked to prioritize for the US.).

Due to the large number of criteria, a partial-profile choice-based conjoint (CBC) survey was developed comprising 14 choice tasks [36], [37]; each choice task presented participants with five disease combinations containing varying levels of 5 of the 21 criteria using an orthogonal experimental design [33]. Participants were asked to select one zoonosis to prioritize for their control and prevention in either Canada or the US (Figure 1). Definitions for the technical terms ‘case-fatality’ (proportion of deaths over the total number of cases) and ‘disease incidence’ (number of new cases of diseases over the last five years in the population) were provided to ensure study participants understood the presented disease criteria. Disease criteria and levels varied between choice tasks and the ordering of the presentation of disease criteria within each choice task was randomized to reduce ordering bias. Two additional fixed choice tasks were included to test the reliability of responses by identifying respondents who did not understand the choice task process and/or fatigue responders. Fixed choice task 1 presented one zoonosis with the highest incidence in humans (10,000 cases), most severe illness in humans (severe clinical symptoms), highest transmission potential between humans (high), highest case-fatality in humans (80%) and the most costly economic burden in humans ($10,000 per sick individual). In comparison, the remaining four zoonoses contained a combination of lower and less severe criteria levels. Fixed choice task 2 presented one zoonosis with the most severe illness in animals (severe clinical symptoms), highest case-fatality in animals (80%), most costly socioeconomic burden in trade in animals (high cost such as culling of herds or destroying infected crops/produce), longest duration of illness in animals (chronic illness or permanent deficits) and rapid change in disease trend in the human population (new emerging disease, rapid increase over the last five years). In comparison, the remaining four zoonoses contained a combination of lower and less severe criteria levels. The fixed choice tasks were also randomized to reduce ordering bias and as an additional measure of reliability. Sawtooth Software CBC module v7 [38] was used to create 300 survey versions, each version presented an efficient experiment design using a balance overlap approach with balanced levels across choice tasks and an orthogonal design [39]. The *D*-efficiency of the experimental design was 908.13326 relative to a full-orthogonal design with a standard error of <0.05 for each criterion level. Sawtooth Software SSI Web v7 [35] was used to randomly assign a survey version to each study participant. Surveys were offered in English, French and Spanish.

### Data Analysis

Hierarchical Bayes (HB) was used to compute parameter estimates (weighted scores) from the CA survey choice data [40]. Sawtooth Software CBC/HB v5.2.8 [2] was used to estimate individual-level parameter estimates (β). The program combined Bayes theorem with a Monte Carlo Markov Chain (MCMC) procedure and the Metropolis/Hasting algorithm to iteratively update parameter estimates drawn from an upper-level model (prior) and a lower-level model (posterior) [40]. The HB algorithm estimates the average parameter estimates for the entire population (prior) and then uses the respondent’s individual data (posterior) to determine how each respondent differed from the population mean. The algorithm will then adjust each respondent’s parameter estimates so that they reflect an optimal mix of the individual respondent choices and the population mean. The optimal mix is determined by the amount of data provided by each respondent (posterior) and the amount of variance in the population mean (prior). The greater the prior variance (the distribution or differences amongst the population), the less Bayesian shrinkage is applied to the mean and the more individuals are allowed to vary such that their parameter estimates provide better individual-level fit to the individual-level responses) [41]. A total of 30,000 preliminary iterations were computed before convergence was observed (graphically and on observing stable goodness-of-fit measures including root-likelihood and variance) and an additional 30,000 iterations were computed per respondent to estimate final parameters. Final parameter estimates (β) are presented as zero-centered standardized utility values by setting the average range of the parameter values of all disease criteria to 100.

Part-worth utility values (β) represent the relative influence each criterion level had on respondent choices with higher values indicating a stronger influence on choice [32]. As part-worth utility values were calculated from choice task sets presenting disease combinations containing varying levels of multiple disease criteria, interaction effects between disease criteria and levels were accounted for in the calculation of part-worth utility values. Twenty-one part-worth utility values, one for each disease criterion, were assigned to the 117 separate disease syndromes by matching the level of each disease criterion to those of disease syndromes [33]. Part-worth utility values were summed up in proportion to the relative frequency of each syndrome within a disease to derive an overall score for each of the 62 diseases. The overall scores were used to rank-order diseases; the higher the score, the higher the ranking on the priority list. As part-worth utility values are interval data, the overall scores cannot be directly compared both within and between countries [32]. Instead, we compare the difference in disease ranking as an overall measure of proximity of diseases both within and between countries. The summed part-worth values approach was used instead of market simulations to apply CA-derived scores to a set of diseases to explore a method comparable to current traditional prioritization methods [14], [19]–[21], [24]–[26].

To estimate the influence of each criterion collectively, importance scores were calculated, for each respondent, as a percentage by dividing the difference in range between the highest and lowest level part-worth utility values by the sum of all part-worth utility value ranges across all criteria, and then averaging those importance scores across respondents. The larger the difference between the levels in a criterion, the higher the importance score and the stronger the influence the criterion had on the decision to prioritize [32]. Individual-level part-worth utility values and importance scores were calculated directly using Sawtooth Software SMRT v4.22.0 [42]. The standard error across individual part-worth utility values was used to quantify uncertainty in the part-worth utility values. *T*-statistics were derived by dividing the mean difference in range in part-worth utility values across each criterion by the standard error of the differences to test each disease criterion for statistical significance in the final model. Overall scores for diseases were calculated as the summation of the mean (population-level) part-worth utility values across the 21 disease criteria. As the overall scores were derived from population-level utility values, uncertainty in the overall scores were quantified by incorporating the standard error across individual part-worth utility values and calculated as the summation of the upper and lower confidence interval part-worth utility values, respectively, across the 21 disease criteria. As part-worth utility values are interval data, we compare changes in disease ranks rather than changes in the overall scores to assess uncertainty.

We used Sawtooth Software CBC/HB v5.2.8 [2] to generate goodness-of-fit measures for the individual-level HB models; these included a percent certainty fit and a root likelihood (RLH). Both of these measures are calculated from the probability of each respondent choosing as they did on each choice task using a logit model estimated with each respondent’s part-worth utility values [40]. The percent certainty and RLH both reflect how well the current model fit is in comparison to a chance model and a perfect model.

Chi Square and Fisher’s exact tests were used to compare the demographic, professional background and survey characteristics of study participants to their respective national populations and to make comparisons by country. The Mann-Whitney test was used to explore differences in completion time by country. National population data for gender, age, region and education were obtained from Statistics Canada [43], [44] and the US Census Bureau [45], [46]. Unpaired *t*-tests, *F*-tests and Welch’s *t*-tests were used to explore differences in standardized importance scores and part-worth utility values between Canada and the US. We used *t*-tests to compare differences in importance scores between the public groups presented in our previous paper [33] and the professional groups presented in this paper, and ANOVA to compare differences between the four groups (Canadian public, Canadian professionals, US public and US professionals). Spearman rank correlation was used to compare disease priority ranks between groups. Friedman’s ANOVA was used to assess uncertainty in the final models on disease priority ranks.

## Results

### Survey and Demographic Characteristics

The response rate for survey completion could not be calculated because the total number of respondents approached at conferences and through advertisement in publications could not be ascertained. Approximately 12,076 Canadians and 33,992 Americans were recruited via direct email invitation, however, as individuals may have been affiliated with one or more professional associations, duplicate posting likely occurred and the exact number of participants approached is unknown. As surveys were collected anonymously, we could not determine survey responses by recruitment mode.

A total of 928 Canadian and 998 US surveys were completed in 13 months and 3 months, respectively, of these, 62 Canadian and 55 US surveys were removed due to not fulfilling the eligibility requirements of being human health or animal health professionals. The majority of Canadian surveys were completed in English (95.5%) while the remaining surveys were completed in French (4.5%). All US surveys were completed in English despite an option to complete the survey in Spanish. Participants passed the survey if all 14 choice task sets were completed and the correct diseases were selected for both fixed choice task set. The fixed choice task questions were designed to present an obvious “best answer” to serve as a screening tool to identify participants who did not understand the choice task process. The Canadian pass rate was 81.6% (707) while the US pass rate was 81.0% (764); there was no significant difference in the pass rate between countries (χ^2^ = 0.1148, *p* = 0.735), nor was there significant difference in the pass rate between Canadian surveys completed in English or French (χ^2^ = 0.1262, *p* = 0.722). Although the fixed choice task sets were introduced to eliminate bias from unreliable respondents, these results suggest that respondents were no more likely to pass or fail (and thus introduce bias) from a particular country or language of completion. The median completion time for passed surveys was 26.9 minutes in Canada and 28.1 minutes in the US; there was no significant difference in the completion time between countries (*p* = 0.515). There were 1,471 completed and passed surveys available for analysis in this study.

There were minor differences between the study populations and their respective national populations by gender, age and geography (Table 1). The most notable difference was a higher educated population in both study populations compared to their respective national populations (*p*<0.001 for both countries). This was expected as the study populations were recruited for their professional background rather than to obtain a representative sample of the national populations. Differences were also observed between the Canadian and US study populations by gender and education (Table 2); there were more female study participants in the US than in Canada (61.2% vs. 54.0%), more high school graduates or less (3.5% vs. 0.6%), Bachelor’s degrees (14.7% vs. 13.5%), Master’s degrees (18.6% vs. 13.6%) and Doctorate degrees (22.7% vs. 20.1%) in the US than in Canada, and more Diplomas, trade or college degrees (3.6% vs. 0.5%) and Professional degrees (48.6% vs. 40.0%) in Canada than in the US.

**Table 1:** Demographic characteristics of Canadian and US study participants in comparison to their respective national population characteristics.

	Canada (n = 707)			US (n = 764)			
	Study Participant	National Population^1^	?^2^		Study Participant	National Population^2^	?^2^
**Gender**			1.75	**Gender**			32.89*
* Male*	46.0%	48.5%			38.8%	49.2%	
* Female*	54.0%	51.5%			61.2%	50.8%	
**Age group**			9.65*	**Age group**			19.59*
* 18 to 34*	26.0%	27.9%			23.9%	30.6%	
* 35 to 50*	34.4%	29.1%			32.6%	27.2%	
* 50+*	39.6%	43.0%			43.5%	42.2%	
**Province**			148.81* +	**Region** 3			7.79
* Alberta*	11.5%	10.6%					
* British Columbia*	8.6%	13.4%		* Midwest*	23.2%	21.7%	
* Manitoba*	6.3%	3.5%		* Northeast*	15.7%	18.3%	
* New Brunswick*	1.4%	2.3%		* South*	34.9%	37.0%	
* Newfoundland and Labrador*	2.0%	1.6%		* West*	26.2%	23.0%	
* Nova Scotia*	2.8%	2.8%					
* Northwest Territories*	0.3%	0.1%					
* Nunavut*	0.0%	0.1%					
* Ontario*	48.9%	38.2%					
* Prince Edward Island*	1.6%	0.4%					
* Quebec*	10.7%	23.9%					
* Saskatchewan*	5.8%	3.0%					
* Yukon*	0.1%	0.1%					
**Educational attainment** 4				**Educational attainment** 5			
* High school graduate or less*	0.6%	45.1%	31012.64*		3.5%	44.5%	11425.27*
* Diploma, trade or college degree*	3.6%	35.1%			0.5%	27.0%	
* Bachelor’s degree*	13.5%	12.7%			14.7%	18.7%	
* Master’s degree*	13.6%	5.8%			18.6%	7.1%	
* Professional degree (MD, DVM* ∧ *)*	48.6%	0.6%			40.0%	1.4%	
* Doctorate degree*	20.1%	0.8%			22.7%	1.3%	

1 2011 population data for individuals 18 years and older in Canada was obtained from Statistics Canada [44].

2 2010 population data for individuals 18 years and older in the US was obtained from the US Census Bureau [46].

3 Regions were:

**Midwest** (Illinois, Indiana, Iowa, Kansas, Michigan, Minnesota, Missouri, Nebraska, North Dakota, Ohio, South Dakota, Wisconsin);

**Northeast** (Connecticut, Maine, Massachusetts, New Hampshire, New Jersey, New York, Pennsylvania, Rhode Island, Vermont);

**South** (Alabama, Arkansas, Delaware, District of Columbia, Florida, Georgia, Kentucky, Louisiana, Maryland, Mississippi, North Carolina, Oklahoma, South Carolina, Tennessee, Texas, Virginia, West Virginia);

**West** (Alaska, Arizona, California, Colorado, Hawaii, Idaho, Montana, Nevada, New Mexico, Oregon, Utah, Washington, Wyoming).

4 2006 education data for individuals 20 years and over in Canada (most current and available data) [43].

5 2010 education data for individuals 18 years and over in the US [45].

* Significant at *p*<0.01.

+ An additional Fisher’s exact test was conducted to take into account of the small sample size in some Provinces and Territories; the results remain unchanged with no statistically significant relationship between the study population and the national population distributions (*p* = 1.000).

∧ MD – Doctor of Medicine degree, DVM – Doctor of Veterinary Medicine degree.

**Table 2:** Demographic characteristics of Canadian and US study participants.

Canada (n = 707)		US (n = 764)		?^2^
**Gender**		**Gender**		15.28*
* Male*	46.0%	* Male*	38.8%	
* Female*	54.0%	* Female*	61.2%	
**Age group**		**Age group**		4.49
* 18 to 34*	26.0%	* 18 to 34*	23.9%	
* 35 to 50*	34.4%	* 35 to 50*	32.6%	
* 50+*	39.6%	* 50+*	43.5%	
**Province**	11.5%	**Region** 1		
* Alberta*				
* British Columbia*	8.6%	* Midwest*	23.2%	–
* Manitoba*	6.3%	* Northeast*	15.7%	
* New Brunswick*	1.4%	* South*	34.9%	
* Newfoundland and Labrador*	2.0%	* West*	26.2%	
* Nova Scotia*	2.8%			
* Northwest Territories*	0.3%			
* Nunavut*	0.0%			
* Ontario*	48.9%			
* Prince Edward Island*	1.6%			
* Quebec*	10.7%			
* Saskatchewan*	5.8%			
* Yukon*	0.1%			
**Educational attainment**		**Educational attainment**		
* High school graduate or less*	0.6%	* High school graduate or less*	3.5%	173.51*
* Diploma, trade or college degree*	3.6%	* Diploma, trade or college degree*	0.5%	
* Bachelor’s degree*	13.5%	* Bachelor’s degree*	14.7%	
* Master’s degree*	13.6%	* Master’s degree*	18.6%	
* Professional degree (MD, DVM* ∧ *)*	48.6%	* Professional degree (MD, DVM^?^)*	40.0%	
* Doctorate degree*	20.1%	* Doctorate degree*	22.7%	

1 Regions were:

**Midwest** (Illinois, Indiana, Iowa, Kansas, Michigan, Minnesota, Missouri, Nebraska, North Dakota, Ohio, South Dakota, Wisconsin);

**Northeast** (Connecticut, Maine, Massachusetts, New Hampshire, New Jersey, New York, Pennsylvania, Rhode Island, Vermont);

**South** (Alabama, Arkansas, Delaware, District of Columbia, Florida, Georgia, Kentucky, Louisiana, Maryland, Mississippi, North Carolina, Oklahoma, South Carolina, Tennessee, Texas, Virginia, West Virginia);

**West** (Alaska, Arizona, California, Colorado, Hawaii, Idaho, Montana, Nevada, New Mexico, Oregon, Utah, Washington, Wyoming).

* Significant at *p*<0.001.

∧ MD – Doctor of Medicine degree, DVM – Doctor of Veterinary Medicine degree.

The Canadian and US study populations differed significantly in professional disciplines, individuals identifying as human health or animal health professionals and by workplace of employment (Table 3). The Canadian study population comprised of more physicians and professionals in the medical sciences (19.9% vs. 10.1%), veterinarians and professionals in the veterinary sciences (34.1% vs. 29.2%) and animal health laboratory technicians (2.0% vs. 0.8%) while the US study population comprised of more epidemiologists (16.0% vs. 10.5%), public health professionals (20.9% vs. 16.4%), infectious disease researchers (8.9% vs. 5.7%), human disease laboratory technicians (1.8% vs. 0.7%) and nurses (4.7% vs. 2.5%). There were more individuals who self-identified as animal health professionals in the Canadian study population, conversely, more individuals self-identified as human health professionals or both human and animal health professionals in the US study population. The Canadian study population included a higher number of professionals working for the government or at hospitals/clinics while the US study population included a higher number of professionals working in academia and from industry. Despite differences in professional background characteristics between Canada and the US, the study populations each reflected a good representation of professional disciplines, animal and human health professionals and workplace of employment within country (Table 3). Consistent between countries was the years in employment with the majority of study participants indicating over 10 years of work experience in their profession (62.5% in Canada, 62.8% in the US).

**Table 3:** Professional background characteristics of Canadian and US study participants.

Canada (n = 707)		US (n = 764)		?^2^
**Professional disciplines**		**Professional disciplines**		
* Epidemiology*	10.5%	* Epidemiology*	16.0%	
* Public Health*	16.4%	* Public Health*	20.9%	
* Physician or Medical Sciences*	19.9%	* Physician or Medical Sciences*	10.1%	
* Infectious Disease Research*	5.7%	* Infectious Disease Research*	8.9%	
* Human Disease Laboratory Technician*	0.7%	* Human Disease Laboratory Technician*	1.8%	131.82*
* Veterinarians and Veterinary Sciences*	34.1%	* Veterinarians and Veterinary Sciences*	29.2%	
* Animal Health Laboratory Technician*	2.0%	* Animal Health Laboratory Technician*	0.8%	
* Nursing*	2.5%	* Nursing*	4.7%	
* Other Profession* 1	8.2%	* Other Profession* 1	6.9%	
* Unknown*	0.0%	* Unknown* 2	0.7%	
**Animal health/Human health**		**Animal health/Human health**		
* Human health*	46.4%	* Human health*	48.5%	
* Animal Health*	43.0%	* Animal Health*	36.8%	16.1*
* Both*	10.6%	* Both*	14.7%	
* Neither*	0.0%	* Neither*	0.0%	
**Years in Employment**		**Years in Employment**		
* Less than 1 year*	3.0%	* Less than 1 year*	3.4%	
* >1 year to 3 years*	8.1%	* >1 year to 3 years*	9.3%	
* >3 years to 5 years*	10.3%	* >3 years to 5 years*	9.8%	4.3
* >5 years to 10 years*	16.0%	* >5 years to 10 years*	14.3%	
* >10 years*	62.5%	* >10 years*	62.8%	
* Unknown*	0.1%	* Unknown*	0.4%	
**Workplace of Employment**		**Workplace of Employment**		
* Academia*	27.2%	* Academia*	30.0%	
* Government*	43.8%	* Government*	40.7%	
* Industry*	7.6%	* Industry*	9.2%	2373.4*
* Hospital/Clinic*	14.6%	* Hospital/Clinic*	10.3%	
* Other* 3	6.2%	* Other* 3	9.2%	
* Unknown*	0.6%	* Unknown*	0.6%	

1 Includes other medical and science related disciplines such as health education, travel medicine, wildlife and aquatic biologists, environmental and ecosystem health, occupational and environmental health and safety, medical entomologists, food inspection and risk assessment, regulatory medicine and policy.

2 This group consisted of five individuals who selected the ‘I prefer not to answer’ response for professional discipline but who identified themselves as either animal health or human health professionals with at least one year of work experience and working in academia, industry or a hospital/clinic.

3 Includes non-government organizations, private consultancy, small businesses, aquariums and zoos, farms, and medical and veterinary associations.

* Significant at *p*<0.001.

### Model Fit

The Canadian model had a percent certainty fit of 83.7% and a root likelihood (RLH) of 0.77, the US model had a percent certainty fit of 84.2% and a root likelihood (RLH) of 0.78. The expected percent certainty for a chance model is 0% and a perfect model is 100% while the expected RLH for a chance model is 0.2 (one divided by five disease combinations per task) and a perfect model is 1.0 [40]. While the models in this study do not represent a perfect model, the models are certainly above satisfactory producing robust part-worth utility values.

### Disease Criteria Importance Scores and Part-worth Utility Values

The importance scores for disease criteria indicate the degree to which each criterion contributed to the decision to prioritize (Table 4). Human-related criteria contributed more to the decision to prioritize than corresponding animal-related criteria with each of the eight matching criteria exhibiting this trend in both countries. The four transmission potential criteria contributed in the following order of preference in both countries: animal-human, human-human, animal-animal and human-animal; thus also revealing a stronger preference for human-related criteria over animal-related criteria. While the contribution of each disease criterion in the decision to prioritize differed with varying degree of importance, ranging from 1.32% to 9.06% (Table 4), each criterion as a whole was statistically significant (*p*<0.05) in the final model for both countries (Table 5).

**Table 4:** Disease criteria importance scores by country.

Disease criteria	Canada (n = 707)			US (n = 764)			*t* 7
	*R* 3	*MS* 4	*SD* 5	*R* 6	*MS*	*SD*	
Incidence of the disease in the last five years (H)^1^	1	8.66	1.94	1	**9.06**	2.21	3.68* ∧
Case-fatality (H)	2	8.07	1.42	2	8.23	1.80	1.89∧
Severity of disease (H)	3	**7.10**	2.08	5	6.49	1.21	6.77* ∧
Disease trend in the last five years (H)	4	6.99	1.46	3	6.97	1.27	0.35∧
Incidence of the disease in the last five years (A)^2^	5	6.47	1.33	4	**6.96**	1.50	6.62* ∧
Economic burden (H)	6	**6.11**	1.57	7	5.84	1.70	3.10* ∧
Duration of illness (H)	7	**5.50**	1.35	8	5.21	1.76	3.52* ∧
Disease trend in the last five years (A)	8	5.48	1.35	6	**5.71**	1.52	3.09* ∧
Transmission potential from animals to humans	9	5.47	1.24	9	5.52	1.31	0.76
Case-fatality (A)	10	**5.26**	1.37	10	5.01	1.37	3.49*
Economic and social burden on trade (A)	11	4.91	1.31	11	**5.20**	1.61	3.75* ∧
Transmission potential between humans	12	4.59	1.06	12	4.72	1.34	2.12∧
Transmission potential between animals	13	3.49	0.88	13	3.46	1.00	0.65∧
Efficacy of control measures (H)	14	3.44	1.73	14	3.56	1.84	1.31
Transmission potential from humans to animals	15	3.34	0.98	16	3.21	1.21	2.29∧
Efficacy of control measures (A)	16	3.20	1.66	15	3.47	1.94	2.87∧
Severity of disease (A)	17	2.90	1.04	17	2.91	0.92	0.28∧
Duration of illness (A)	18	2.77	1.00	18	2.59	0.86	0.35∧
How much is known scientifically about the disease	19	2.63	1.54	20	2.53	1.28	1.28∧
High-risk groups (H)	20	2.13	0.90	19	2.03	0.83	2.26
High-risk groups (A)	21	**1.49**	0.73	21	1.32	0.70	4.72*

1 (H) = human-related characteristic, for example, case-fatality in *humans*.

2 (A) = animal-related characteristic, for example, case-fatality in *animals*.

3 Relative rank of disease criteria by importance scores for Canadian participants; the Table is presented in order of importance for Canadian participants.

4 Mean importance score across respondents.

5 Standard deviation of importance scores across respondents.

6 Relative rank of disease criteria by importance scores for US participants.

7 *t*-statistic; *d.f*. = 1,469.

* *p*<0.0024 (Bonferroni-corrected *p*-value cut-off derived from *p* = 0.05/21).

∧ Adjusted for unequal variance (identified by the *F*-test of equality of variances) using the Welch *t*-test; Satterthwaite’s *d.f.* = 1,117.19 to 1,468.99.

Scores in **bold** indicate disease criteria with statistically significant difference in importance scores between Canada and the US; scores for the country with the highest score (i.e. placed more importance on) are in **bold**.

**Table 5:** Disease criteria and standardized part-worth utility values for disease criteria levels by country.

Disease criteria^1^ and corresponding levels	Canada			US				*t* ^5^
	MUV(*β*)^2^	*LCL* ^3^	*UCL* ^4^	MUV(*β*)^2^		*LCL* ^3^	*UCL* ^4^	
*Incidence of the disease in the Canadian/US human population in the last five years*								
0 cases	−84.29	−85.75	−82.84	−88.49	−90.22		−86.77	3.65*** ∧
5 cases	−39.26	−40.79	−37.72	−37.28	−38.36		−36.21	2.07* ∧
100 cases	27.40	26.42	28.38	24.96	24.06		25.85	3.61***
10,000 cases	96.15	94.12	98.18	100.82	98.92		102.71	3.29**
*Case-fatality in humans*								
No deaths or deaths are rarely reported	−75.39	−76.68	−74.10	−80.16	−81.56		−78.77	4.92*** ∧
Case-fatality is low (6%)	−43.64	−45.02	−42.26	−43.15	−44.51		−41.80	0.49
Case-fatality is moderate (35%)	26.78	25.72	27.84	33.50	32.21		34.79	7.89*** ∧
Case-fatality is high (80%)	92.25	90.86	93.63	89.81	87.87		91.75	2.00* ∧
*Severity of illness in humans*								
No clinical symptoms or illness that is not noticeable	−71.85	−73.53	−70.17	−69.07	−70.12		−68.02	2.75** ∧
Mild clinical symptoms (time off work, some medical assistanceand personal care at home)	−29.02	−30.26	−27.77	−21.36	−22.41		−20.30	9.23*** ∧
Moderate clinical symptoms (urgent medical care and hospital admission)	25.35	24.31	26.40	23.96	23.17		24.76	2.07* ∧
Severe clinical symptoms (failure of major organ system/s necessitatinglong-term hospital admission)	75.51	73.56	77.47	66.46	65.27		67.65	7.76*** ∧
*Disease trend in Canada/US in the last five years in humans*								
Decline over the last five years	−74.57	−76.18	−72.95	−71.46	−72.58		−70.33	3.10** ∧
Stable over the last five years	−29.32	−30.73	−27.90	−28.76	−29.79		−27.73	0.63∧
Increase over the last five years	34.32	32.75	35.88	26.22	25.19		27.24	8.48*** ∧
New emerging disease, rapid increase over the last five years	69.57	68.25	70.88	74.00	72.78		75.21	4.87***
*Incidence of the disease in the Canadian/US animal population in the last five years*								
0 cases	−61.74	−62.85	−60.63	−64.46	−65.65		−63.27	3.27** ∧
5 cases	−29.20	−30.20	−28.20	−31.43	−32.69		−30.18	2.72** ∧
100 cases	17.36	16.50	18.22	16.90	15.98		17.81	0.73∧
10,000 cases	73.58	72.25	74.91	79.00	77.28		80.71	4.89*** ∧
*Economic burden in humans*								
No cost to the health care system and individuals	−61.33	−62.83	−59.82	−58.33	−59.91		−56.75	2.70** ∧
Low cost ($100 per sick individual)	−19.17	−20.37	−17.97	−22.53	−23.55		−21.51	4.18*** ∧
Moderate cost ($1,000 per sick individual)	16.08	14.96	17.19	19.97	19.03		20.91	5.24*** ∧
High cost ($10,000 per sick individual)	64.42	62.82	66.02	60.89	59.23		62.54	3.01** ∧
*Duration of illness in humans*								
No illness observed or only a few days of illness	−49.67	−51.16	−48.17	−50.87	−52.43		−49.31	1.09∧
Short-term illness (weeks)	−23.54	−24.83	−22.25	−14.86	−15.88		−13.85	10.36*** ∧
Medium−term illness (months)	11.37	10.07	12.68	10.36	9.54		11.18	1.29∧
Chronic illness (years) or illness with permanent deficits	61.83	60.42	63.23	55.37	53.64		57.10	5.68*** ∧
*Disease trend in Canada/US in the last five years in animals*								
Decline over the last five years	−57.17	−58.41	−55.92	−58.34	−59.75		−56.93	1.22∧
Stable over the last five years	−24.70	−25.79	−23.62	−25.27	−26.26		−24.28	0.76
Increase over the last five years	27.70	26.58	28.81	24.47	23.55		25.38	4.38*** ∧
New emerging disease, rapid increase over the last five years	54.17	52.70	55.64	59.14	57.80		60.48	4.91***
*Transmission potential from animals to humans*								
No transmission from animals to humans	−51.13	−52.41	−49.84	−55.81	−57.11		−54.51	5.01***
Low transmission from animals to humans	−30.90	−31.91	−29.88	-26.35	−27.41		−25.30	6.08*** ∧
Moderate transmission from animals to humans	22.35	21.38	23.31	24.46	23.54		25.38	3.10**
High	59.68	58.42	60.93	57.71	56.55		58.87	2.26*
*Case-fatality in animals*								
No deaths or deaths are rarely reported	−47.01	−48.25	−45.78	−43.05	−44.07		−42.04	4.86*** ∧
Case-fatality is low (6%)	−32.72	−33.77	−31.67	−31.44	−32.52		−30.37	1.66
Case-fatality is moderate (35%)	20.45	19.16	21.74	17.90	16.75		19.05	2.89** ∧
Case-fatality is high (80%)	59.29	57.82	60.75	56.60	54.96		58.23	2.40* ∧
*Economic and social burden on trade in animals*								
No cost to trade in animals	−39.39	−40.44	−38.34	−40.98	−42.42		−39.53	1.74∧
Low cost to trade in animals (vaccination of herds)	−31.14	−32.40	−29.88	−29.35	−30.62		−28.08	1.95
Moderate cost to trade in animals (restriction of movement and trade)	12.11	11.13	13.09	8.38	7.40		9.37	5.25***
High cost to trade in animals (culling of herds or destroying infected crops/produce)	58.42	56.99	59.84	61.94	60.34		63.55	3.22** ∧
*Transmission potential between humans*								
No transmission between humans	−41.55	−42.71	−40.40	−43.37	−44.75		−41.99	1.97* ∧
Low transmission between humans	−27.46	−28.54	−26.38	−25.59	−26.71		−24.47	2.36* ∧
Moderate transmission between humans	18.12	17.02	19.21	17.42	16.37		18.48	0.89
High transmission between humans	50.90	49.96	51.83	51.54	50.41		52.66	0.86∧
*Transmission potential between animals*								
No transmission between animals	−29.36	−30.43	−28.30	−32.72	−33.73		−31.71	4.50***
Low transmission between animals	−21.22	−22.22	−20.22	−15.98	−16.75		−15.20	8.10*** ∧
Moderate transmission between animals	13.24	12.37	14.11	11.46	10.49		12.43	2.67** ∧
High transmission between animals	37.35	36.48	38.21	37.23	36.37		38.09	0.18
*Efficacy of control measures in humans*								
Highly effective in reducing disease burden	8.92	5.90	11.93	1.68	−1.56		4.91	3.21** ∧
Moderately effective in reducing disease burden	8.69	7.38	10.01	10.40	9.33		11.47	1.97* ∧
Minimally effective in reducing disease burden	−8.66	−10.49	−6.83	−1.76	−3.42		−0.10	5.48***
Not effective at all in reducing disease burden	−8.95	−11.49	−6.41	−10.32	−12.78		−7.86	0.76
*Transmission potential from humans to animals*								
No transmission from humans to animals	−30.91	−32.23	−29.59	−27.71	−28.85		−26.57	3.59*** ∧
Low transmission from humans to animals	−17.02	−18.08	−15.97	−17.78	−18.94		−16.63	0.95∧
Moderate transmission from humans to animals	15.71	14.68	16.75	14.47	13.35		15.59	1.60∧
High transmission from humans to animals	32.22	31.36	33.08	31.02	29.93		32.12	1.68∧
*Efficacy of control measures in animals*								
Highly effective in reducing disease burden	19.40	17.02	21.78	20.90	18.49		23.31	0.86
Moderately effective in reducing disease burden	12.96	11.42	14.49	10.04	8.35		11.73	2.50** ∧
Minimally effective in reducing disease burden	−15.21	−17.03	−13.39	−11.28	−12.97		−9.59	3.11**
Not effective at all in reducing disease burden	−17.15	−19.28	−15.02	−19.67	−22.12		−17.21	1.52∧
*Severity of illness in animals*								
No apparent clinical signs or the animal-source of infection is non-living (e.g. food-source)	−23.16	−23.96	−22.35	−26.28	−27.36		−25.21	4.58*** ∧
Mild clinical signs (minor distress such as fever, lethargy, shivering, constipation, loose feces)	−16.48	−17.51	−15.45	−13.28	−14.23		−12.34	4.49***
Moderate clinical signs (moderate distress such as difficult breathing, bleeding from openings, aborted fetuses)	8.70	7.71	9.70	12.19	11.07		13.31	4.57*** ∧
Severe clinical signs (severe distress such as convulsion, organ failure, neurological involvement)	30.94	29.69	32.18	27.38	26.45		28.30	4.49*** ∧
*Duration of illness in animals*								
No illness observed or only a few days of illness	−18.01	−19.39	−16.63	−20.65	−21.78		−19.52	2.91** ∧
Short-term illness (weeks)	−11.57	−12.89	−10.25	−8.07	−8.91		−7.23	4.38*** ∧
Medium-term illness (months)	3.03	1.94	4.13	3.29	2.38		4.20	0.35∧
Chronic illness (years) or illness with permanent deficits	26.54	25.46	27.62	25.44	24.45		26.43	1.48
*How much is known scientifically about the disease*								
Knowledge of the disease is well known and scientifically valid	0.63	−2.17	3.42	−12.47	−14.81		−10.13	7.05*** ∧
Knowledge of the disease exists but the validity of the information is uncertain	2.78	1.88	3.68	7.74	7.03		8.46	8.44*** ∧
Knowledge of the disease is currently insufficient	3.14	1.90	4.38	7.82	6.63		9.01	5.33***
There is no scientific knowledge of the disease	−6.55	−8.20	−4.90	−3.09	−4.47		−1.72	3.16**
*High risk groups in humans*								
No	−18.95	−19.82	−18.09	−17.46	−18.39		−16.52	2.30* ∧
Unknown	−3.10	−3.92	−2.27	−2.23	−3.12		−1.35	1.40∧
Yes	22.05	21.11	23.00	19.69	18.88		20.51	3.71*** ∧
*High risk groups in animals*								
No	−9.53	−10.39	−8.67	−9.76	−10.49		−9.03	0.40∧
Unknown	−2.92	−3.80	−2.04	−0.88	−1.53		−0.23	3.66*** ∧
Yes	12.45	11.59	13.31	10.64	9.80		11.49	2.94**

1 Presented in order of importance to Canadians. ^2^Mean part-worth utility values (β) across respondents.^ 3^95% lower confidence interval (LCL) of mean part-worth utility values (β) across respondents. ^4^95% Upper confidence interval (LCL) of mean part-worth utility values (β) across respondents^ 5^ *t*-statistic; *d.f*. = 1,469.

* *p*<0.05,

** *p*<0.01,

*** *p*<0.001.

∧ Adjusted for unequal variance (identified by the *F*-test of equality of variances) using the Welch *t*-test; Satterthwaite’s *d.f.* = 1,176.87 to 1469.

Although differences were observed between countries, both groups considered *incidence of the disease in the last five years in humans* and *case-fatality in humans* to be the most influential criteria in the decision to prioritize zoonoses (Table 4). Similarly, both groups considered *high-risk groups in animals* to be the least influential criteria in the decision to prioritize zoonoses. An additional eight disease criteria were ranked equally between the countries (*transmission potential from animals to humans*, *case-fatality in animals*, *economic and social burden on trade in animals*, *transmission potential between humans*, *transmission potential between animals*, *efficacy of control measures in humans*, *severity of disease in animals* and *duration of illness in animals*) while the remaining criteria differed only by a maximum of two ranked positions (*severity of the disease in humans*, *disease trend in the last five years in humans*, *disease incidence in the last five years in animals*, *economic burden in humans*, *duration of illness in humans*, *disease trend in the last five years in animals*, *transmission potential from humans to animals*, *efficacy of control measures in animals*, *how much is known scientifically about the disease* and *high risk groups in humans*) indicating a general consensus between the two countries on the contribution of the 21 disease criteria in the decision to prioritize zoonoses.

The part-worth utility values (β) indicate the relative influence each level had on respondent choices with higher values representing a stronger degree of influence on choice. The mean part-worth utility values and the upper and lower uncertainty estimates of mean part-worth utilities values are presented in Table 5. The wider the range in part-worth utility values between the lowest levels and highest levels within each criterion, the more influence the criterion had on the decision to prioritize. There were nine disease criteria in which the importance score differed significantly between countries (Table 4); the utility trends for these criteria can be broadly summarized as follows:

Canadian professionals were more strongly influenced by severity of disease in humans, economic burden in humans, duration of illness in humans, case-fatality in animals and high-risk groups in animals (p<0.0024 for all, Table 4). US professionals were more strongly influenced by incidence of the disease in the last five years in humans, incidence of the disease in the last five years in animals, disease trend in the last five years and economic and social burden on trade in animals (p<0.0024 for all, Table 4). Despite the differences in importance scores, there was agreement between countries on the levels of least importance (lowest part-worth utility values) and levels of highest importance (highest part-worth utility values) for each of these nine disease criteria, with incremental increases in the part-worth utility values for the levels in between (Table 5). While the strength of preference in disease criteria importance scores and part-worth utility values differed between countries (Tables 4 and 5), there was general agreement in the contribution of disease criteria in the decision to prioritize zoonoses. The difference may be due to a difference in the perceived threat of disease and disease characteristics by country.

### Disease Priority Lists

Table 6 presents the final ranking of diseases derived from their overall CA scores. The range in the overall scores by diseases differed between Canada and the US and correlates with the part-worth utility values derived from the country-specific models (Table 5). Canadians considered rabies to be the most important zoonoses to prioritize, followed by Nipah virus encephalitis, H1N1 influenza, variant Creutzfeldt-Jakob disease and listeriosis. These were also the top five priority diseases in the US, ranked in different order. There was also consensus between the bottom five diseases on the priority list with three of the five least important diseases appearing in both priority lists. Although differences were observed in disease rankings between countries, the majority of diseases (76%) were within ten ranked positions of each other indicating general agreement in disease ranks between countries (Spearman’s rho = 0.8356, *p* = 0.000).

**Table 6:** Disease priority list by country (Canadian professionals vs. US professionals).

Canada	score	rank	US	score	rank	Difference in rank (relative to Canada)
Rabies	278.10	1	variant Creutzfeldt-Jakob disease (CJD)	351.60	1	3
Nipah virus encephalitis	246.68	2	Rabies	281.37	2	−1
Influenza (H1N1)	227.82	3	Influenza (H1N1)	252.12	3	0
variant Creutzfeldt-Jakob disease (CJD)	210.21	4	Nipah virus encephalitis	225.35	4	−2
Listeriosis	198.56	5	Listeriosis	220.26	5	0
Ebola virus haemorrhagic fever	180.87	6	Babesiosis	173.62	6	42*
Marburg haemorrhagic fever	170.29	7	Ebola virus haemorrhagic fever	165.23	7	−1
Influenza (H5N1)	121.41	8	Anaplasmosis	159.34	8	36*
Leishmaniasis	48.82	9	Marburg haemorrhagic fever	153.39	9	−2
Botulism	45.17	10	Tularemia	144.94	10	5
Cryptosporidiosis	26.17	11	Paralytic shellfish poisoning	129.00	11	20*
Salmonellosis	15.21	12	Influenza (H5N1)	110.63	12	−4
Hendra virus	14.42	13	Hantavirus pulmonary syndrome	93.64	13	3
*Escherichia coli* infection	0.41	14	Plague	87.04	14	9
Tularemia	−0.63	15	American trypanosomiasis (Chagas disease)	78.04	15	5
Hantavirus pulmonary syndrome	−2.49	16	Q fever	73.52	16	2
Chlamydiosis	−8.92	17	Rocky Mountain spotted fever	70.98	17	12*
Q fever	−18.05	18	Shigellosis	60.62	18	4
Giardiasis	−18.39	19	Brucellosis	60.26	19	5
American trypanosomiasis (Chagas disease)	−25.83	20	*Escherichia coli* infection	59.23	20	−6
Leptospirosis	−43.06	21	Botulism	56.69	21	−11*
Shigellosis	−43.94	22	Leptospirosis	45.42	22	−1
Plague	−45.41	23	Cryptosporiodiosis	33.81	23	−12*
Brucellosis	−59.44	24	Eastern equine Encephalitis	33.62	24	4
Crimean-Congo hemorrhagic fever	−60.52	25	Leishmaniasis	30.51	25	−16*
Psittacosis/Avian chlamydiosis	−63.85	26	Salmonellosis	29.32	26	−14*
Toxoplasmosis	−67.15	27	Chlamydiosis	17.26	27	−10
Eastern equine encephalitis	−79.02	28	Hendra virus	14.19	28	−15*
Rocky Mountain spotted fever	−81.03	29	Campylobacteriosis	−11.02	29	7
Bartonellosis	−89.51	30	Giardiasis	−13.19	30	−11*
Paralytic shellfish poisoning	−103.81	31	Lyme Disease	−29.38	31	3
West Nile virus	−112.52	32	Psittacosis/Avian chlamydiosis	−31.17	32	−6
Powassan virus	−115.83	33	Toxoplasmosis	−44.74	33	−6
Lyme disease	−118.77	34	Typhus	−70.57	34	11*
Echinococcosis	−120.87	35	Bartonellosis	−74.08	35	−5
Campylobacteriosis	−125.73	36	West Nile virus	−75.08	36	−4
Toxocariasis	−141.46	37	Crimean-Congo hemorrhagic fever	−80.23	37	−12*
Anthrax	−143.42	38	Powassan virus	−88.11	38	−5
Cutaneous larva migrans	−157.06	39	Coccidioidomycosis	−106.80	39	20*
Baylisascariasis	−183.52	40	Toxocariasis	−116.22	40	−3
Old/New World screwworm	−186.96	41	Anthrax	−117.18	41	−3
Severe Acquired Respiratory Syndrome	−199.66	42	Echinococcosis	−120.47	42	−7
Western equine encephalitis	−213.41	43	Cutaneous larva migrans	−124.71	43	−4
Anaplasmosis	−217.35	44	Western equine Encephalitis	−128.69	44	−1
Typhus	−220.03	45	Cysticerosis/Taeniasis	−134.82	45	12*
Trichinosis	−222.20	46	Baylisascariasis	−163.90	46	−6
Japanese encephalitis	−241.55	47	Japanese encephalitis	−170.38	47	0
Babesiosis	−255.06	48	Old/New World screwworm	−193.85	48	−7
Lassa fever	−256.97	49	Hepatitis A	−202.62	49	7
Rift Valley fever	−294.15	50	Venezuelan equine Encephalitis	−209.34	50	3
Cholera	−296.07	51	Severe Acquired Respiratory Syndrome	−210.28	51	−9
Monkeypox	−299.54	52	Trichinosis	−263.98	52	−6
Venezuelan equine Encephalitis	−305.15	53	Lassa fever	−267.09	53	−4
Yellow fever	−339.74	54	Rift Valley fever	−274.87	54	−4
Bovine tuberculosis	−340.22	55	Monkeypox	−281.64	55	−3
Hepatitis A	−352.26	56	Yellow Fever	−285.72	56	−2
Cysticerosis/Taeniasis	−414.42	57	Cyclosporiasis	−337.63	57	1
Cyclosporiasis	−466.51	58	Bovine tuberculosis	−355.90	58	−3
Coccidioidomycosis	−472.26	59	Dengue fever	−399.17	59	1
Dengue fever	−525.52	60	Cholera	−405.05	60	−9
La Crosse encephalitis	−630.24	61	St. Louis encephalitis	−417.89	61	1
St. Louis encephalitis	−663.97	62	La Crosse encephalitis	−445.96	62	−1

* Diseases that deviated by more than 10 ranked positions between countries.

As we included a broad group of zoonotic diseases, many of these diseases may not be relevant for specific stakeholder groups, for example, there may be groups only interested in vector-borne diseases, food-borne and enteric diseases, exotic diseases, endemic diseases or diseases affecting certain commodity groups. The priority list can be further broken down into these sub-groups and the priority diseases by subgroup include: vector-borne diseases (leishmaniasis, Chagas disease and the plague in Canada; babesiosis, anaplasmosis and the plague in the US), food-borne and enteric diseases (variant Creutzfeldt-Jakob disease, listeriosis and botulism in Canada; variant Creutzfeldt-Jakob disease, listeriosis and paralytic shellfish poisoning in the US), exotic diseases (Nipah virus encephalitis, Ebola virus haemorrhagic fever and Marburg haemorrhagic fever for both Canada and the US) and endemic diseases (rabies, H1N1 influenza and variant Creutzfeldt-Jakob disease in Canada; variant Creutzfeldt-Jakob disease, rabies and H1N1 influenza in the US) (Table 6).

Diseases of high priority exhibited high incidence (H/A), high case-fatality (H/A), severe symptoms in humans, an increasing or emerging trend (H/A), high socioeconomic burden (H/A), prolonged duration of illness in humans and high transmission potential from animals to humans. However, it was not necessary to exhibit each of these characteristics to be identified as a priority (for example, H1N1 influenza and babesiosis both have low case-fatality rates while Nipah virus encephalitis, Ebola virus hemorrhagic fever and Marburg hemorrhagic fever do not occur naturally in North America). Diseases of low priority generally included rare diseases or diseases with a large proportion of asymptomatic cases (H/A), low case-fatality (H/A), mild symptoms in humans, stable or decreasing trend (H/A), low socioeconomic burden (H/A), short duration of illness in humans and low transmission potential from animals to humans.

Canadians considered leishmaniasis, Hendra virus, salmonellosis, cryptosporidiosis and Crimean-Congo hemorrhagic fever of higher priority than Americans (difference of 12 ranked positions or more, Table 6). Conversely, Americans considered babesiosis, anaplasmosis, paralytic shellfish poisoning, coccidioidomycosis, cysticercosis and Rocky Mountain spotted fever of higher priority than Canadians (difference of 12 ranked positions or more, Table 6). This can be explained by regional differences in both human and animal disease incidence; for example, babesiosis does not occur naturally in Canada but is endemic in the US [47], anaplasmosis is extremely rare in Canada but endemic in the US [48] and Rocky Mountain spotted fever is found only in Western Canada but is distributed throughout the US [49]. Regional differences in disease trend also likely contributed to different rankings, for example, paralytic shellfish poisoning has been increasing in the US [50] but is stable in Canada. Other differences can be explained by differences in part-worth utility values in disease criteria by country (Table 5).

To test the uncertainty in the part-worth utility values derived from the statistical models, we applied the 95% lower and upper confidence intervals of the mean part-worth utility values (Table 5) to compare the ranking of diseases. There was no statistical differences in the ranking of the diseases when uncertainty estimates were applied with 58 of 62 disease ranks unchanged and 4 diseases changing by one rank in Canada (Friedman’s χ^2^ = 0.000, *p* = 1.000) and 53 of 62 disease ranks unchanged, 8 diseases changing by one rank and one disease changing by two ranks in the US (Friedman’s χ^2^ = 0.008, *p* = 0.996) (S2). There were also no statistical differences in the ranking of the diseases when uncertainty estimates were applied to cluster of similar diseases (vector-borne diseases, food-borne and enteric diseases, exotic diseases and endemic diseases) (*p*>0.993 for all disease groups for both Canada and the US).

### Comparison between Public and Professional Groups

Results from the same survey administered to the public were published previously [33]. The Canadian and US public groups completed the survey faster with a lower pass rate compared to their respective professional groups (Table 7). There was no difference in the gender and age distribution between the Canadian public and Canadian professional groups, but there were a higher number of study participants from Alberta, Manitoba, Ontario and Saskatchewan in the professional group. There was no difference in the geographic distribution between the US public and the US professionals, but there were a higher number of middle to older age females amongst the study participants. This may reflect the target population in the professional group. A highly educated population was observed in both professional groups compared to the public groups despite the public groups already representing a highly educated population in comparison to the national populations [33].

**Table 7:** Survey and demographic comparison between the public and professional groups by country.

CANADA				US				
	Public (n = 761)	Professionals (n = 707)	?^2^			Public(n = 778)	Professionals (n = 764)	?^2^
**Completion time (minutes)**	18.3	26.9	−			21.6	28.1	−
**Pass rate** 1	58.2%	76.2%	79.94*			59.4%	76.6%	74.87*
**Gender**			1.12	**Gender**				26.9*
* Male*	48.0%	46.0%				48.2%	38.8%	
* Female*	52.0%	54.0%				51.8%	61.2%	
**Age group**			1.32	**Age group**				15.87*
* 18 to 34*	27.3%	26.0%				29.7%	23.9%	
* 35 to 50*	35.1%	34.4%				27.5%	32.6%	
* 50+*	37.5%	39.6%				42.8%	43.5%	
**Province**			114.71* +	**Region** 2				5.24
* Alberta*	10.6%	11.5%						
* British Columbia*	13.1%	8.6%		* Midwest*		22.6%	23.2%	
* Manitoba*	3.8%	6.3%		* Northeast*		18.1%	15.7%	
* New Brunswick*	2.1%	1.4%		* South*		35.9%	34.9%	
* Newfoundland and Labrador*	1.4%	2.0%		* West*		23.4%	26.2%	
* Nova Scotia*	2.8%	2.8%						
* Northwest Territories*	0.1%	0.3%						
* Nunavut*	0.0%	0.0%						
* Ontario*	38.9%	48.9%						
* Prince Edward Island*	0.9%	1.6%						
* Quebec*	22.7%	10.7%						
* Saskatchewan*	3.0%	5.8%						
* Yukon*	0.4%	0.1%						
**Educational attainment**				**Educational attainment**				
* High school graduate or less*	34.8%	0.6%	6482.59*		42.9%		3.5%	6023.43*
* Diploma, trade or college degree*	25.4%	3.6%			4.5%		0.5%	
* Bachelor’s degree*	27.1%	13.5%			35.0%		14.7%	
* Master’s degree*	7.4%	13.6%			13.0%		18.6%	
* Professional degree (MD, DVM)*	3.3%	48.6%			2.8%		40.0%	
* Doctorate degree*	1.5%	20.1%			1.8%		22.7%	

1 Participants passed the survey if all 14 choice task sets were completed and the correct diseases were selected for both fixed choice task set.

2 Regions were:

**Midwest** (Illinois, Indiana, Iowa, Kansas, Michigan, Minnesota, Missouri, Nebraska, North Dakota, Ohio, South Dakota, Wisconsin);

**Northeast** (Connecticut, Maine, Massachusetts, New Hampshire, New Jersey, New York, Pennsylvania, Rhode Island, Vermont);

**South** (Alabama, Arkansas, Delaware, District of Columbia, Florida, Georgia, Kentucky, Louisiana, Maryland, Mississippi, North Carolina, Oklahoma, South Carolina, Tennessee, Texas, Virginia, West Virginia);

**West** (Alaska, Arizona, California, Colorado, Hawaii, Idaho, Montana, Nevada, New Mexico, Oregon, Utah, Washington, Wyoming).

* Significant at *p*<0.001.

+ An additional Fisher’s exact test was conducted to take into account of the small sample size in some Provinces and Territories; the results remain unchanged with no statistically significant relationship between the study population and the national population distributions (*p* = 1.000).

The percent certainty for the Canadian and US public models were 79.4% each [33]. The models presented here for the Canadian and US professionals indicate the professional models were better fitted than the public models (83.7% and 84.2%, respectively), though models of percent certainty of 70% or higher are regarded as models of good fit [51].

**Figure 2:**
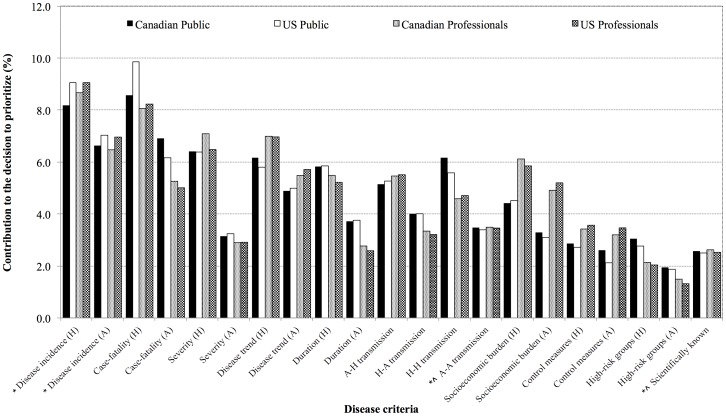
Mean disease criteria importance scores by country and by public and professional groups. Disease criteria are presented in order of the human-related criteria with the highest mean score across all four groups, followed by the corresponding animal-related criteria. (*Disease criteria with mean scores that did not differ significantly between combined public and combined professional groups (*p*>0.0024 - Bonferroni-corrected *p*-value cut-off) using *t*-tests. ^∧^Disease criteria with mean scores that did not differ significantly across all four groups (*p*>0.0024 - Bonferroni-corrected *p*-value cut-off) using ANOVA. All remaining disease criteria had mean scores that were significantly different between combined public and professionals groups (*p*<0.0024) and across all four groups (*p*<0.0024)).

The mean disease criteria importance scores by country and by groups are presented in Figure 2. Similarities were observed between the combined public groups and combined professional groups while differences were observed between the public and professional groups. We identified differences in mean scores between the public and the professional groups for each disease criterion (*p*<0.0024) with the exception of *disease incidence in humans, disease incidence in animals*, *animal-animal transmission potential* and *how much is known scientifically about the disease*. The public groups considered criteria relating to the individual-level of disease burden such as *case-fatality* (H/A), *human-to-human transmission* and *duration of illness* (H/A) more important in the decision to prioritize (*p*<0.0024) while the professional groups deemed criteria relating to the societal and population-level of disease burden such as *socio-economic burden* (H/A), *disease trend* (H/A) and *efficacy of control measures* (H/A) to be more important (*p*<0.0024).

A unique priority list was derived for each group in each country (Tables 6 and 8). Although differences were observed, similarities were also apparent in diseases in the top 10 and in the bottom 10. Seven of the top ten diseases were common across all groups (rabies, Nipah virus encephalitis, Ebola virus hemorrhagic fever, Marburg hemorrhagic fever, H1N1 influenza, variant Creutzfeldt-Jakob disease *and* listeriosis) while five of the bottom ten diseases were common across all groups (bovine tuberculosis, cyclosporiasis, Dengue fever, La Crosse encephalitis and St. Louis encephalitis). Despite more similarities in demographics, model fit, disease criteria importance scores and part-worth utility values between the two public and the two professional groups, there was more uniformity across priority lists between the Canadian public and Canadian professionals (Spearman’s rho = 0.9707, *p* = 0.000) and between the US public and US professionals (Spearman’s rho = 0.9774, *p* = 0.000) than between the Canadian and US public groups (Spearman’s rho = 0.8497, *p* = 0.000) and between the Canadian and US professional groups (Spearman’s rho = 0.8356, *p* = 0.000).

**Table 8:** Disease priority list by country (Canadian public vs. US public).

Canada	score	rank	US	score	rank	Difference in rank (relative to Canada)
Nipah virus encephalitis	284.01	1	variant Creutzfeldt-Jakob disease (CJD)	368.89	1	5
Rabies	280.02	2	Rabies	295.44	2	0
Ebola virus haemorrhagic fever	260.24	3	Nipah virus encephalitis	286.10	3	−2
Marburg haemorrhagic fever	225.13	4	Ebola virus haemorrhagic fever	276.87	4	−1
Influenza (H1N1)	208.70	5	Marburg haemorrhagic fever	250.86	5	−1
variant Creutzfeldt-Jakob disease (CJD)	194.02	6	Influenza (H1N1)	207.22	6	−1
Listeriosis	177.78	7	Listeriosis	200.75	7	0
Hendra virus	64.79	8	Tularemia	164.88	8	4
Influenza (H5N1)	64.69	9	Anaplasmosis	137.19	9	36*
Salmonellosis	37.65	10	Hantavirus pulmonary syndrome	106.09	10	10*
Leishmaniasis	23.44	11	Paralytic shellfish poisoning	104.85	11	25*
Tularemia	10.33	12	Babesiosis	90.74	12	38*
*Escherichia coli* infection	−8.46	13	American trypanosomiasis (Chagas disease)	81.17	13	5
Cryptosporiodiosis	−11.29	14	Plague	79.65	14	9
Eastern equine Encephalitis	−26.50	15	Hendra virus	65.12	15	−7
Botulism	−33.51	16	Influenza (H5N1)	62.25	16	−7
Shigellosis	−36.76	17	Shigellosis	55.89	17	0
American trypanosomiasis (Chagas disease)	−52.78	18	Eastern equine Encephalitis	54.28	18	−3
Giardiasis	−54.12	19	Leishmaniasis	53.60	19	−8
Hantavirus pulmonary syndrome	−59.94	20	Salmonellosis	47.74	20	−10*
Campylobacteriosis	−60.02	21	*Escherichia coli* infection	38.07	21	−8
Toxoplasmosis	−60.58	22	Q fever	19.95	22	5
Plague	−62.54	23	Cryptosporiodiosis	10.44	23	−9
Psittacosis/Avian chlamydiosis	−74.75	24	Rocky Mountain spotted fever	7.94	24	9
Leptospirosis	−79.55	25	Botulism	−26.23	25	−9
Chlamydiosis	−79.67	26	Campylobacteriosis	−27.72	26	−5
Q fever	−94.88	27	Leptospirosis	−32.95	27	−2
West Nile virus	−109.20	28	Lyme Disease	−45.26	28	2
Bartonellosis	−114.43	29	Brucellosis	−47.38	29	5
Lyme Disease	−124.52	30	Chlamydiosis	−52.67	30	−4
Crimean-Congo hemorrhagic fever	−130.11	31	Psittacosis/Avian chlamydiosis	−53.33	31	−7
Powassan virus	−142.24	32	Toxoplasmosis	−58.94	32	−10*
Rocky Mountain spotted fever	−145.83	33	Giardiasis	−70.07	33	−14*
Brucellosis	−149.39	34	Powassan virus	−84.47	34	−2
Anthrax	−167.66	35	West Nile virus	−85.51	35	−7
Paralytic shellfish poisoning	−170.06	36	Bartonellosis	−94.16	36	−7
Echinococcosis	−180.88	37	Typhus	−103.02	37	9
Toxocariasis	−183.97	38	Coccidioidomycosis	−109.79	38	20*
Cutaneous larva migrans	−199.71	39	Crimean-Congo hemorrhagic fever	−132.98	39	−8
Lassa fever	−203.02	40	Anthrax	−144.16	40	−5
Baylisascariasis	−219.41	41	Echinococcosis	−147.03	41	−4
Severe Acquired Respiratory Syndrome	−222.05	42	Baylisascariasis	−155.26	42	−1
Old/New World screwworm	−245.17	43	Toxocariasis	−157.14	43	−5
Western equine Encephalitis	−250.06	44	Cutaneous larva migrans	−158.61	44	−5
Anaplasmosis	−256.71	45	Cysticercosis/Taeniasis	−168.25	45	12*
Typhus	−272.70	46	Western equine Encephalitis	−185.49	46	−2
Japanese encephalitis	−273.33	47	Severe Acquired Respiratory Syndrome	−194.66	47	−5
Monkeypox	−279.78	48	Hepatitis A	−205.98	48	6
Trichinosis	−316.26	49	Japanese encephalitis	−230.44	49	−2
Babesiosis	−316.48	50	Lassa fever	−231.04	50	−10*
Venezuelan equine Encephalitis	−329.70	51	Old/New World screwworm	−251.11	51	−8
Yellow Fever	−330.35	52	Monkeypox	−274.35	52	−4
Cholera	−342.29	53	Venezuelan equine Encephalitis	−279.00	53	−2
Hepatitis A	−359.51	54	Yellow Fever	−303.53	54	−2
Bovine tuberculosis	−370.50	55	Trichinosis	−338.97	55	−6
Rift Valley fever	−372.81	56	St. Louis encephalitis	−363.37	56	6
Cysticercosis/Taeniasis	−443.42	57	Cyclosporiasis	−363.46	57	2
Coccidioidomycosis	−459.90	58	La Crosse encephalitis	−394.32	58	3
Cyclosporiasis	−490.94	59	Bovine tuberculosis	−397.95	59	−4
Dengue fever	−520.64	60	Cholera	−416.70	60	−7
La Crosse encephalitis	−589.41	61	Dengue fever	−422.66	61	−1
St. Louis encephalitis	−597.52	62	Rift Valley fever	−425.87	62	−6

* Diseases that deviated by more than 10 ranked positions between countries.

## Discussion

Zoonotic diseases are a significant public health burden in North America [6]–[9]. As there are limited resources available for their control and prevention, a scientifically driven framework for the prioritization of zoonoses is essential. An increasing number of recent studies have focused on quantitative methods for disease prioritization [13], [14], [21], [24]–[26], though there is currently no agreeable standard. In this study, we present on the novel use of the quantitative method, CA, for prioritizing zoonoses in North America. Our method fits a statistical model to choice data under a robust experimental design to generate relative weighted scores for disease criteria and levels. This overcomes the primary limitation of current methods that rely on simplified linear point-scoring systems without weights [16]–[20] or an expert Delphi panel to assign subjective weights that are applied to linear point-scoring systems [13], [14], [21], [24], [26]. Further, CA allows for disease criteria to be considered jointly, acknowledging dependence between criteria. CA forces individuals to prioritize on the basis of scientific information, eliminating biases associated with disease names and as individuals are provided with all the information necessary to prioritize, the method allows for the inclusion of individuals with no prior knowledge of the diseases (public) or health professionals who may not be knowledgeable in the full range of diseases.

In comparison to other prioritization studies, this study included a larger number of disease criteria than previously used [14], [16]–[25], although many of these studies involved diseases exclusively in humans hence our animal-related criteria would not have been appropriate in these studies. We also used a broad objective for disease prioritization, asking our respondents to prioritize diseases for their control and prevention without specifying the method of control and prevention (for example, regulation, management, vaccination, laboratory diagnosis, research and surveillance). Many studies focused on a specific aspect of disease prioritization, for example, prioritizing solely for surveillance [19], [20] or prioritizing a specialized group of diseases, for example, emerging or food-borne zoonoses only [24], [25]; these studies would require fewer disease criteria to assess diseases. Our study also included the largest number of professional participants of any disease prioritization study [14], [16]–[26]; this was feasible due to the novel CA method used in which respondents were presented with all the information necessary to prioritize diseases. This allowed for wide participation including health professionals who may not be familiar with the full range of diseases being prioritized, but could add much value to the decision to prioritize due to their specific professional training and experience. While our method involves more work and a larger study population than traditional methods, the use of CA overcomes traditional limitations in prioritization methods including simplified linear scores, subjective weights, the assumption of independence between disease criteria, biases associated with disease names and limited expert participation.

Our previous study indicated individuals with no prior knowledge of infectious diseases were capable of producing meaningful results with satisfactory model fits [33]. This study suggests professionals with knowledge or experience in prioritizing zoonoses were capable of producing meaningful results with better-fitted models than the general public. Disease criteria importance scores were realistic and sensible, consistent with findings from previous prioritization studies [13], [14], [21], [24]–[26]. Part-worth utility values demonstrated face-validity with higher preferences given to higher levels and lower preferences to lower levels. The disease priority lists generated from applying CA-derived part-worth utility values to diseases produced a list of diseases that are reasonable for prioritization, particularly when further divided into subgroups such as vector-borne diseases, food-borne and enteric diseases, exotic diseases and endemic diseases. Analysis of the uncertainty estimates in the part-worth utility values showed that disease priority lists did not change substantially; indicating good model fits producing robust part-worth utility values.

Our study found that the public groups placed more importance on disease criteria relating to the individual-level of disease burden while the professional groups placed more importance on disease criteria relating to the population-level of disease burden. Our previous study on the focus groups conducted to inform this current study affirms this observation with similar findings between the public and professional focus groups [12]. Despite closer resemblances in demographics, model fit and disease criteria importance and part-worth utility values between the combined public and combined professional groups, there was more unity in the disease priority lists between the Canadian public and Canadian professionals and between the US public and US professionals than the combined respective public and professional groups. This suggests that regional differences in country-specific disease criteria (disease incidence and trend) contributed more to disease priority rankings than differences in demographics and disease criteria preference. This finding is not surprising given study participants were asked to prioritize diseases for their control and prevention in their respective countries, moreover, if disease incidence was high and disease trend was emerging in the US but the disease was not found in Canada, the part-worth utility values assigned to the disease in Canada would reflect the lowest levels despite disease incidence and disease trend having high importance to Canadians, while the part-worth utility values assigned in the US would reflect the highest levels. This would explain why diseases such as anaplasmosis, babesiosis, paralytic shellfish poisoning and Rocky Mountain spotted fever were ranked much higher by the US groups than their respective groups in Canada.

Limitations related to the study were outlined in the previous paper [33]. Additional limitations associated with the study presented in this paper relate to the multiple recruitment methods used to recruit professionals. It is unknown whether some recruitment methods resulted in better-quality responses than others; we assume study participants responded to the survey in the same manner regardless of their mode of recruitment. However, if a particular recruitment method resulted in lower-quality survey responses, inconsistent responders would have been screened out with the two fixed choice task questions. Multiple recruitment methods may have also resulted in a different group of qualified professionals within the study population; nonetheless, the demographic data collected suggest the two professional groups were representative of individuals with some prior knowledge of infectious diseases. Further, in comparison to the public groups, the two professional groups were distinct populations with higher education and a background in an eligible professional discipline.

Although the survey was offered in three languages (English, French and Spanish) across the two countries, surveys were only completed in English and French. While we found no significant difference in the pass rate between the surveys completed in English and French, it is unknown whether translation bias may have resulted in differences in responses between languages or whether bias was introduced as a result of having no surveys completed in Spanish. An additional uncertainty that cannot be account for in this study is the quality and/or the lack of data in the literature used to assign levels of criteria to diseases [33]. It is unknown to what degree this type of uncertainty may have affect the presented results, however, the best available data available at the time of analysis was used [33] and as better quality data and more scientific information about the diseases becomes available, the priority lists presented in this study can only be improved upon.

As noted in our previous paper, there are multiple objectives for prioritizing zoonoses (for example, research, regulation, control, prevention, management, vaccination, diagnosis, cost-effective and surveillance), although study participants were asked to prioritize for policy implementation for the control and prevention of zoonoses, they may have prioritized with another objective in mind. This would be even more apparent in the professional groups who are actively involved in public health and zoonoses. There is no way to measure this type of bias and we assume participants were consistent in their prioritization objectives. Additionally, our professional study population included a wide range of professional disciplines, yet not all disciplines can be treated equally. For example, medical doctors and veterinarians are more likely to have better knowledge and experience with zoonotic diseases compared to laboratory technicians, thus, treating our professional study population uniformly may weaken differences across different groups of professionals. An argument could also be made that some professional disciplines in our study may not truly reflect experts in zoonotic diseases, for example public health nurses, despite the fact that the professionals selected in our study were distinct from individuals in the general public and that as nurses usually represent the front line of defense during an outbreak, their experience would add much value to the decision to prioritize. The same argument could be made for laboratory technicians, who may not be experts in zoonotic diseases, but have a strong understanding on the identification and diagnosis of disease-causing pathogens. This raises the question of which experts should be engaged in prioritizing zoonotic diseases for public health. While our study results represent the collective opinion of a broad group of experts across multiple disciplines, we did identify important differences between our public and professional groups. Our future research goal will be to investigate how individuals from different professional disciplines may prioritize zoonotic diseases differently and explore the issue of which individuals should be responsible for making such decisions.

We present the first zoonoses prioritization exercise involving public health, veterinary and medical professionals in North America. Our novel quantitative approach is an established method in other disciplines. Our study results were validated with satisfactory model fits and reasonable disease criteria scores and part-worth utility values. These results illustrate that CA can be used as a potential tool for the prioritization of zoonoses, particularly as a method to overcome subjective weighting and scoring of disease criteria. Other limitations that can be addressed by CA include assuming independence between disease criteria; biases associated with disease names; and limited expert participation. Although this approach involves more work and a larger study population, disease priority lists can be revised on a regular basis by updating criteria levels to match the most current disease trends, thus, a large-scale CA study can be conducted to establish the baseline disease criteria scores with minor year-to-year updates of disease priority lists. This type of scientifically driven framework for disease prioritization would be of value in North America.

## Supporting Information

File S1 **Professional associations, publications and conferences targeted for professional study participants recruitment.**
(DOCX)

File S2 **Disease rank comparison.**
(XLSX)
