# Supplementary material for: A Quantitative Approach to the Prioritization of Zoonotic Diseases in North America: A Health Professionals’ Perspective
Source: PLoS One. 2013 Aug 21;8(8):e72172. doi: 10.1371/journal.pone.0072172 (PMC3749166; doi:10.1371/journal.pone.0072172)
Supplement: File S1 — Professional associations, publications and conferences targeted for professional study participants recruitment. (DOCX) [file pone.0072172.s001.docx]

**Direct email - Professional Associations**

Academy of Rural Veterinarians, Academy of Veterinary Consultants

Agricultural Research Service – United States Department of Agriculture

American Association of Bovine Practitioners

American Association of Small Ruminant Practitioners

Association of Medical Microbiology and Infectious Disease Canada

Association of Veterinary Epidemiology and Preventative Medicine

Association of Veterinary Technician Educators

Canadian Association of Swine Veterinarians

Canadian Cooperative Wildlife Health Centre

Canadian Integrated Program for Antimicrobial Resistance Surveillance

Canadian Public Health Association

Conference of Research Workers in Animal Diseases

Evidence-Based Veterinary Medical Association

Food Safety Research and Response Network

International Society for Infectious Diseases (ProMED mailing list)

National Agricultural Biosecurity Center – Kansas State University

One Health Initiative

Ontario Veterinary College

United States Animal Health Association.

**Advertisement in publications**

- Canadian Nursing Association
- Canadian Veterinary Medical Association
- Ontario Agency for Health Protection and Promotion – Regional Infection Control Network.

**Conferences**

3^rd^ North American Congress in Epidemiology,

American Public Health Association

American Society for Microbiology

Canadian Public Health Association

Canadian Veterinary Medical Association

Ontario Veterinary Medical Association

The Ontario Public Health Convention

United States Animal Health Association

Care was taken to ensure that conference participants were provided only as much information in person as other study participants recruited via direct email or advertisement.
